# Possible Advantages of S53P4 Bioactive Glass in the Treatment of Septic Osteoarthritis of the First Metatarsophalangeal Joint in the Diabetic Foot

**DOI:** 10.3390/jcm10061208

**Published:** 2021-03-15

**Authors:** Matevž Kastrin, Vilma Urbančič Rovan, Igor Frangež

**Affiliations:** 1 Department of Surgical Infections, University Medical Center Ljubljana, Zaloska Cesta 2, 1000 Ljubljana, Slovenia; matevz.kastrin@kclj.si; 2 Department of Endocrinology, Diabetes and Metabolic Diseases, University Medical Centre Ljubljana, Zaloska Cesta 2, 1000 Ljubljana, Slovenia; vilma.urbancic@kclj.si; 3 Faculty of Medicine, University of Ljubljana, Vrazov Trg 2, 1000 Ljubljana, Slovenia

**Keywords:** septic osteoarthritis, diabetic foot, bioactive glass, foot ulcer, segmental resection of the first MTP joint, diabetic foot osteomyelitis

## Abstract

Biomechanically, the great toe with its metatarsophalangeal (MTP) joint plays a key role in standing and walking, making the first MTP joint one of the main predilection sites for ulcer formation, and consequently for bone and joint infection and even amputation. If conservative treatment fails, the main goal of surgery is to remove all infected tissue and preserve the first ray. To improve surgical outcomes, development of new biomaterials like Bioactive Glass S53P4 has begun. Bioactive Glass is useful because of its antibacterial properties; furthermore, its osteostimulative and osteoconductive qualities make the bone substitute particularly suitable as a bone defect filler for the treatment of osteomyelitis. The aim of our retrospective observational study was to compare the outcomes following ulcerectomy with segmental resection of the infected joint and bone and temporary stabilization with an external fixator, both with and without added Bioactive Glass. A comparison of added Bioactive Glass with the traditional surgical treatment in septic osteoarthritis of the first MTP joint showed Bioactive Glass to be effective. During a one-year follow-up, patients with Bioactive Glass required no additional antibiotic therapy or surgical intervention. Bioactive Glass, when applied to the diabetic foot, showed itself to be a safe bone substitute biomaterial.

## 1. Introduction

Biomechanically, the great toe with its metatarsophalangeal (MTP) joint plays a key role in standing and walking. In patients with diabetes, pathological changes in biomechanics occur, such as atrophy of intrinsic muscles, fibrosis, and distalization of fat pads which normally relieve the pressure under the metatarsal heads. Due to sensory neuropathy, such patients do not feel increased pressure in these points. This leads to a redistribution of pressure while standing and walking, making the first MTP joint one of the main predilection sites for ulcer formation. Furthermore, the broken skin barrier allows for colonization and consequent infection of soft tissues or further penetration of the infection into the joint and bone [1].

No consensus exists in the literature on the advantages of surgical over conservative (antibiotic) therapy in the treatment of such infections. Patients with diabetes and infected ulcers involving the MTP joint and periarticular bone of the great toe are very often difficult to treat, particularly when they insist on being treated conservatively and refuse any surgical procedure.

However, the standard protocol in these cases consists of extensive debridement of the infected soft tissues and resection of the infected bone [2]. This approach, especially when a large area is affected, could make the great toe unstable, deformed, or even nonfunctional. Amputation of the first ray significantly changes the weight-bearing pattern of the foot, and could alter the balance of the forefoot’s intrinsic musculature, leading to lesser toe deformity [3]. According to Lavery, patients develop increased pressure in the forefoot and heel after a first ray amputation, and have an increased rate of lesser toe deformities compared to the contralateral foot [4]. The recurrence rate of osteomyelitis is high; in some cases, a relapse occurs after just a few months. Based on these findings, it is understandable why patients after great toe or first ray amputation need another, higher reamputation. Murdoch et al. reported the reamputation rate after hallux or first ray amputation to be possibly as high as 60% [5].

Several alternative treatment methods have been suggested, with the main goal of performing salvage treatment of the first ray and avoiding complications such as increased deformity, ulceration, and subsequent amputation. Chan et al. [6] described a simple resection of the first MTP joint through a dorsal approach with closure over drains. Firstly, the mobile flail joint quickly fills up with granulation and fibrous tissue, thus still allowing a reasonable range of movement. On the other hand, Roukis and Landsman performed a two-stage operation: resection of all infected bone, placement of antibiotic-loaded polymethylmethacrylate beads and an external fixator in the first stage, and subsequent MTP arthrodesis with an iliac crest graft in the second stage. The same researchers later performed a single-stage procedure using antibiotic-loaded cement to fill the defect after extensive debridement and resection of the affected bone [2].

The most common germs involved in diabetic foot infections are gram-positive bacteria, especially Staphylococcus aureus. As infected ulcers complicated by osteomyelitis often require prolonged antibiotic therapy, this can induce the development of methicillin-resistant S. aureus (MRSA) and/or cause side effects, so further treatment must be stopped [7]. In order to reduce the use of antibiotics in the treatment of osteomyelitis, development of new biomaterials and bone substitutes began. At the moment, the third generation of biomaterials, which stimulate tissue regeneration and repair by gene activation properties, is in use. One of them is S53P4 Bioactive Glass (BG), invented by Lerry Hench at the University of Florida in 1969 [8]. In 2011, the EU approved the usage of S53P4 BG as a specific option in the treatment of osteomyelitis. It is useful because of its antibacterial properties; furthermore, its osteostimulative and osteoconductive qualities make the bone substitute particularly suitable as a bone defect filler for the treatment of osteomyelitis [9].

However, only individual cases of S53P4 BG use in combination with surgical treatment of diabetic foot infections can be found in the literature [9,10]. The aim of our retrospective study was to evaluate the possible advantages of segmental resection of the first MTP joint in combination with S53P4 BG compared to segmental resection without bone substitutes.

## 2. Patients and Methods

### 2.1. Study Design

The study was conducted as an observational retrospective study among patients who were treated at the Department of Surgical Infections at the University Medical Centre Ljubljana for plantar or marginal-medial ulcers and with osteomyelitic involvement of the first MTP joint from 1 January 2019 to 15 November 2019. Twenty-two patients met the inclusion criteria (Table 1).

### 2.2. Inclusion and Exclusion Criteria

The inclusion criteria were as follows: Adult patients (age > 18 years) with diabetes type 1 or 2, with a neuropathic plantar or marginal-medial ulcer and with osteomyelitic involvement of the first MTP joint. None of the included patients showed signs of improvement in the healing of the foot ulcer within six weeks despite optimal management following the latest Guidelines from the International Working Group on the Diabetic Foot (IWGDF) [11]. Osteomyelitis was confirmed by a positive probe-to-bone test and X-ray (the presence of lytic lesions and/or a periosteal reaction) of the foot [11].

Patients with extensive diabetic foot infections (extensive involvement of soft tissues contraindicating conservative treatment with an indication for a minor amputation, phlegmon, or extension of the infection to the midfoot), Charcot neuroarthropathy, systemic signs of inflammation, hepatic impairment, and known allergies to antibiotics were excluded from the study. In addition, patients with significant peripheral arterial disease (PAD) (absence of peripheral pulses and ankle brachial index (ABI) < 0.9), in whom revascularization was indicated due to critical ischemia, were also excluded.

### 2.3. Surgical Technique

The surgical procedure was ulcerectomy with segmental resection of the infected joint and bone and temporary stabilization with an external fixator, with the main goal of promoting wound healing, removing the focus of infection, stabilizing the medial column, and reducing the risk of repeated ulceration of the first ray and the lesser rays. With this procedure, appropriate debridement of the infectious tissues can be achieved, and the first ray and its potential contribution to weight-bearing are simultaneously preserved [2,3].

**Figure 1:**
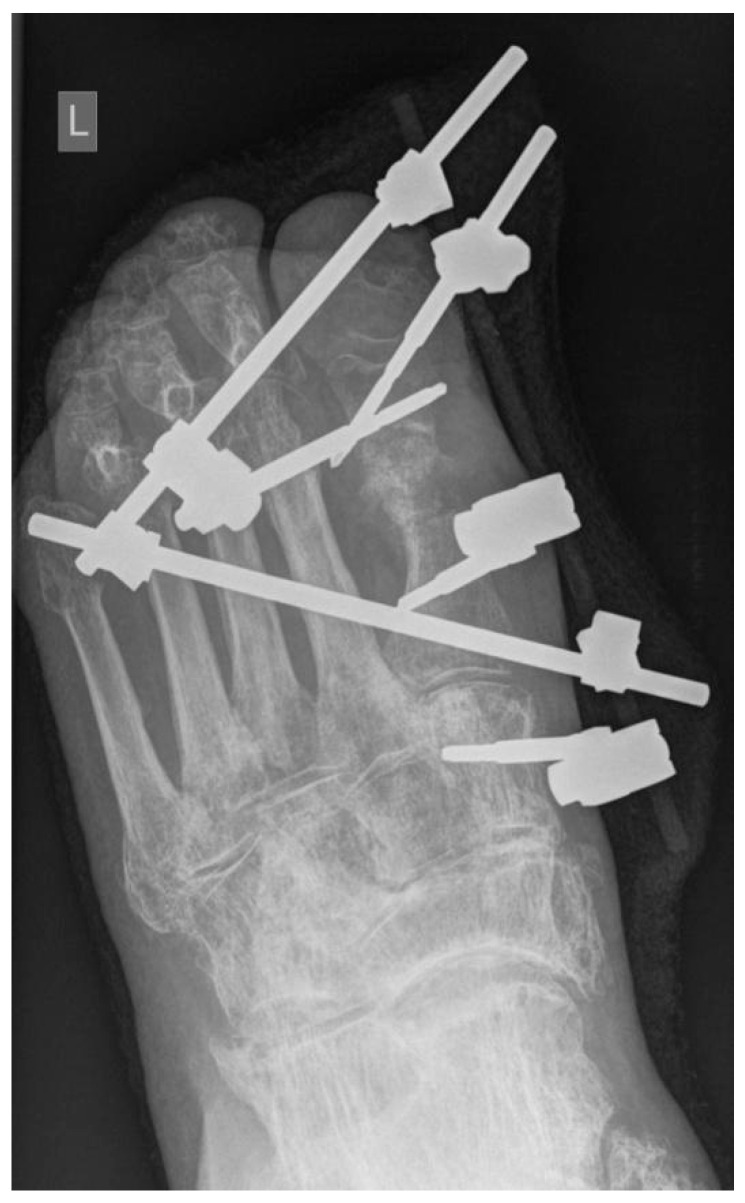
Segmental resection of the first MTP joint with Bioactive Glass S53P4; MTP: metatarsophalangeal.

**Figure 2:**
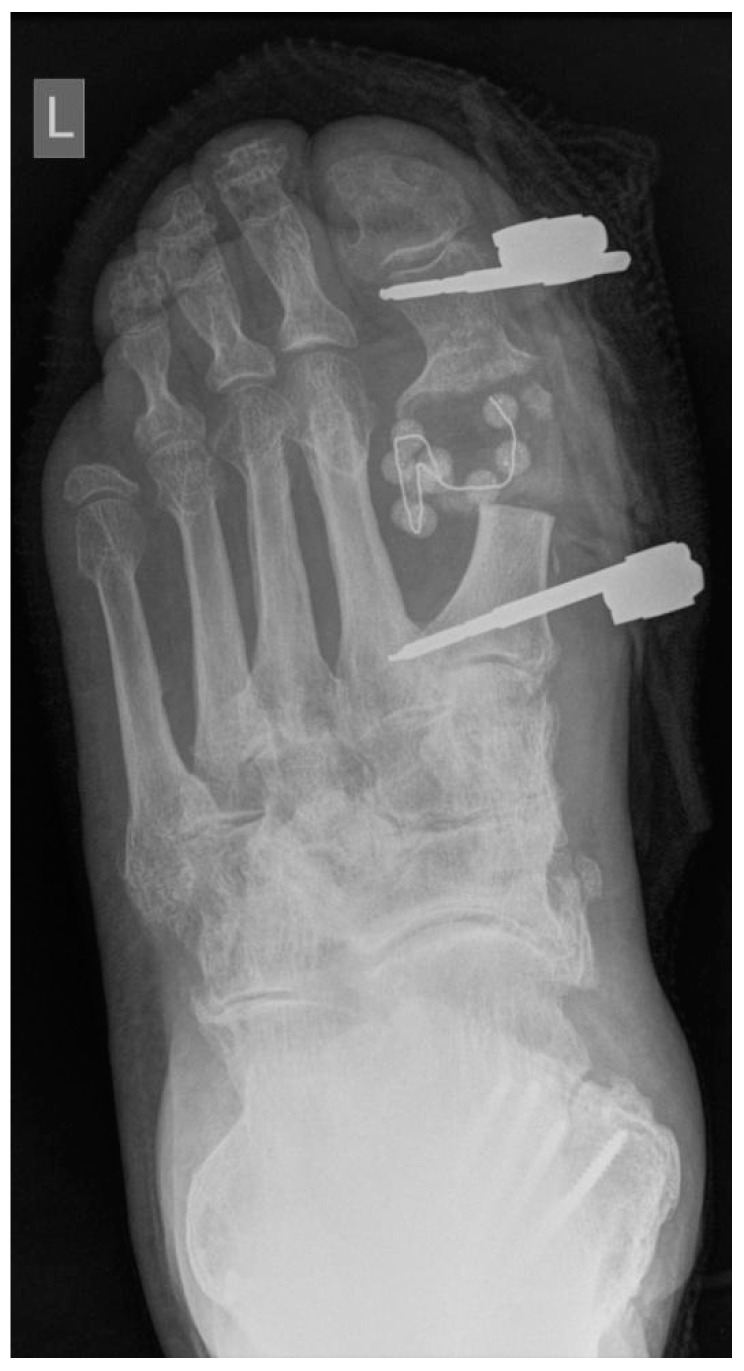
Segmental resection of the first MTP joint with a Septopal^®^ Chain.

Of the 22 patients with septic osteoarthritis, 10 (45.5%) (group A) were treated with segmental resection of the first MTP joint and periarticular bone, stabilization with an external fixator, and a local application of biomaterial S53P4 BG mixed with 5 mL of venous blood (Figure 1). Group B included 12 patients (54.5%) who were treated with segmental resection, temporary application of a Septopal^®^ Chain (Zimmer Biomet Deutschland GmbH, Freiburg im Breisgau, Deutschland) of 10 beads into the void with each bead containing 7.5 mg gentamicin sulphate, and stabilization with an external fixator (Figure 2). All patients were operated on by the same surgeon. Group A had a one-stage procedure, and group B, a two-stage procedure to remove the Septopal^®^ beads after three weeks. Patients in both groups were operated on in a spinal block.

All surgical procedures were done through one incision (carefully protecting the digital neurovascular bundle) that included the excision of the ulcer. Through this incision, the segmental resection of the infected joint and adjacent infected bone using an oscillating saw was performed, including removal of sesamoid bones. Tissue samples of the soft and bone tissues were sent for histology and microbiology evaluation (Table 2) that confirmed osteomyelitis in all cases.

The lavage of the wound was made with 100 mL of sodium hypochlorite and at least 1000 mL of normal saline solution. After segmental resection, a Kirschner wire was inserted under X-ray control for temporary stabilization of the first ray. Then the first ray was stabilized with a mini external fixator to retain the full length of the first ray. From that stage, the surgical procedures differ. In group A, the cavity remaining after debridement and segmental resection was filled with S53P4 BG granules. In group B, the cavity was filled with a Septopal^®^ Chain of 10 beads. Finally, in both groups, soft tissues and overlying skin were primarily closed with a single layer of wide nylon vertical mattress sutures. At the end of the procedure, a tendo-Achilles lengthening was performed using the triple hemisection percutaneous technique in patients who lacked at least five degrees of passive ankle joint dorsiflexion with the knee in extension. It has been shown that Achilles tendon lengthening can significantly reduce the risk of recurrent diabetic foot ulceration [12].

During the lesion healing, all patients received specific temporary postoperative off-loading forefoot healing shoes for the operated foot and an elevation sole for the contralateral foot to offset the imbalance.

A basal X-ray of the foot was performed on the second postoperative day to evaluate the correct position of the external fixator in all patients, and the correct position of the S53P4 BG in group A.

Postoperatively, all patients were given empiric parenteral antibiotic therapy with amoxicillin/clavulanic acid, 1.2 g twice a day, which was modified as soon as the results of microbiological tests were available. A total of 14 days of parenteral antibiotic therapy was achieved in all patients during hospitalization. After discharge into home care, specific peroral antibiotic therapy was prolonged for four weeks according to consensus on the surgical aspect of managing osteomyelitis in the diabetic foot [13,14,15].

Three weeks after surgery, an X-ray was repeated in both groups. At this time, sutures were removed in group A. In group B, the Septopal^®^ beads were removed at the second part of the surgical procedure through the same surgical incision. In group B, the sutures were removed three weeks later.

Six weeks after surgery, the X-ray was repeated in group A and the external fixator was removed as an outpatient procedure. In group B, the X-ray was repeated after eight weeks (after the first surgery) and the external fixator was removed, also as an outpatient procedure.

After removal of the external fixator, patients in both groups were provided with customized diabetic shoes with a rocker sole made on the basis of individual plaster foot castings, in accordance with consensus recommendations on advancing the standard of care for treating neuropathic foot ulcers in patients with diabetes [11].

Patients were evaluated using a clinical exam, serum test for inflammation, and an X-ray after 3, 6, and 12 months, postoperatively.

### 2.4. Outcome Measures

The main outcome was complete resolution of septic osteoarthritis and osteomyelitis after the described procedure that results in complete healing without any additional procedure.

Secondary outcomes included the need for an additional cycle of antibiotic therapy in the period following the primary surgical intervention, and the need for a new surgical intervention—amputation of the great toe. The safety outcomes assessed were fever and an allergic reaction. For group A with implanted S53P4 BG, a possible foreign-body reaction was also monitored. In case of osteomyelitic focus recurrence, we looked for possible new lesions on any part of the foot in the following 12 months through periodic X-rays of the foot. When signs of inflammation or infection appeared, the values of laboratory parameters of inflammation were checked.

The study was conducted in accordance with the Helsinki Declaration on medical research on humans and good clinical practice. All patients were informed about the performed procedure and provided signed informed consent.

### 2.5. Statistical Analysis

Collected data were presented as means +/- SD for continuous variables and as percentages for categorical variables. For statistical analysis, the paired Student’s t-test for normally distributed variables and the Mann-Whitney U test for skewed variables were used. The Chi-square test was used to compare categorical variables between groups and, in case of small frequencies, the Fisher exact test was used. Odds ratios and a 95% confidence interval (95% CI) were calculated. The significance level was set at *p* < 0.05. Statistical analysis was performed using the statistical package for social science software (SPSS), v.25 (IBM Corp, Armonk, NY, USA).

## 3. Results

All 22 patients were postoperatively monitored with clinical evaluation and with X-rays (as described previously, at 3 weeks, then 2, 3, 6, and 12 months after surgery) for healing evaluation and possible osteomyelitis recurrence. Successful healing with a complete resolution of osteomyelitis was achieved in all 10 patients from group A and in 9/12 patients from group B (*p* = 0.221).

In two patients from group B, early postoperative healing was not satisfying.

In one patient from group B, phlegmon of the great toe developed three weeks after surgery, requiring TMT amputation of the great toe.

The second patient from group B had an infection with Methicillin Resistant Staphylococcus Aureus and refused prolonged hospitalization and intravenous antibiotic treatment. He was discharged with peroral antibiotic treatment, which was not sufficient to prevent amputation of the great toe.

One patient from group B had an uneventful postoperative course until 11 months after surgery. He developed valgus deformity of the great toe, and consequently an ulcer on the medial site of the first MTP joint. There was no sign of osteomyelitis in the X-ray, CT scan, or tissue samples of the bone. The wound was closed surgically; the valgus deformation was treated with a silicone spacer.

Two additional patients from group B developed valgus deformation of the great toe without recurrence of an ulcer which was observed at a six-month postoperative check-up. Both were managed with silicone spacers.

Patients were also monitored for possible adverse reactions to foreign material used to fill the bone defect. None of the patients developed a fever or an allergic reaction. In group A, with implanted S53P4 BG, no clinical or radiologic signs of foreign body reaction were observed.

## 4. Discussion

Diabetic foot is one of the most dangerous complications of diabetes mellitus. According to the literature, up to 30% of people with diabetes will develop a foot ulcer, and every 20 s, an amputation of part of a limb or a whole limb is conducted globally due to diabetes [11]. Diabetes prevalence has increased dramatically in the developed world. Once affecting mainly old and bedridden ischemic patients, there are nowadays more and more young patients with diabetes to whom we have the responsibility of restoring an acceptable quality of life after the resolution of an acute episode. With this in mind, several new alternative ways of treatment with the main goal of performing salvage treatment of the first ray have been developed, as conservatively as possible to preserve short- and long-term quality of life and, most importantly, to reduce the risk of iatrogenic recurrences [10]. Years ago, Murdoch presented the importance of great toe preservation [5]. He reported a series of 71 patients who had undergone a disarticulation at the first MTP joint or who had needed an even more proximal amputation. Of these 71 patients, 40 (56%) subsequently required more proximal amputation of the first ray within 10 years, revealing the importance of preserving the first ray. Another important study by Bowker [16] demonstrated that extensive first ray amputation with removal of most of the first metatarsal was devastating to proper foot function owing to the loss of the medial column, which is essential for both stance and forward progression. Another potential benefit of maintaining a stabilized, though shortened, great toe is preservation of the medial buttressing effect of the great toe to help prevent imbalance of the forefoot’s intrinsic musculature and the secondary varus angulation of the second toe’s MTP joint, frequently seen after first ray amputation [2].

Our study presents the results of septic osteoarthritis treatment with adjacent osteomyelitis of the first MTP joint using segmental resection and stabilization with an external fixator. The benefit of the described approach is the preservation of the first ray. The same surgical technique was evaluated by Dalla-Paola et al. [2]. They presented a series of 28 patients who had the same surgical treatment as our patients in group B. In their study, only three patients (10.71%) underwent surgical reoperation due to ulceration relapse in the first ray, while in our study, only one patient (4.55%) was managed with additional soft tissue surgery 11 months after the first operation because of ulcer development caused by valgus deformation (without infection of the bone). Two additional patients in group B developed valgus deformation without consequences to the soft tissue or ulcer recurrence. This implies that the described procedure with temporary stabilization of the resected first MTP joint with an external fixator is effective in treating ulcers with septic osteoarthritis and, more importantly, preserving the first ray.

With its antibacterial, osteostimulative, and osteoconductive properties, Bioactive Glass S53P4 enables modification of the described operation from a two-step to a single-step procedure. Few studies on the use of S53P4 BG in the surgical treatment of diabetic foot complications can be found in the literature, and none of them exclusively describes septic osteoarthritis of the first MTP joint.

De Giglio et al. [9] performed an observational retrospective study and compared surgical debridement of osteomyelitis of the foot with and without added S53P4 BG. One difference between their and our study is that ours only included cases with septic osteoarthritis of the first MTP joint with adjacent bone osteomyelitis, while they presented cases of different locations of foot osteomyelitis. The second difference is that they removed the osteomyelitic bone with a sharp spoon and added S53P4 BG, while we performed a segmental resection to remove the whole segment of infected bone tissue. In De Giglio’s study, successful resolution of osteomyelitis in the group that received S53P4 BG was 90%, compared to 100% in ours. The better results in our study could be attributed to a more accurate segmental resection compared to removing osteomyelitic soft bone with a sharp spoon.

Another publication presenting the use of S53P4 BG in diabetic foot surgery was published by Iacopy et al. [10]. They enrolled 10 patients who received S53P4 BG in addition to standard care during the surgical procedure. In six weeks of follow-up, 8 out of 10 patients healed (80%). The difference between Iacopi’s study and ours is that they enrolled a wide variety of patients (including seven that needed percutaneous transluminal angioplasty) and surgical procedures, while our patients had no significant impairment of macrocirculation and all underwent the same type of surgical procedure.

The use of S53P4 BG in our study and in the previous two studies resulted in no adverse reactions and no fever or allergic reactions. Therefore, S53P4 BG seems to be an effective and safe bone substitute material.

In conclusion, our retrospective observational study performed in a group of diabetic patients with a plantar ulcer and osteomyelitic involvement of the first MTP joint has shown that segmental resection of the affected joint and bone (and application of S53P4 BG into the void) provides healing in a high percentage of cases. During a one-year follow-up, the number of recurrent ulcers and surgical re-interventions was low. Although bone loss occurred as a result of the operations, our surgical treatment can still be defined as conservative, in contrast to amputation. The latter not only creates the conditions resulting in a greater risk of recurrent ulcers, but also represents a potentially greater risk of a second, more proximal, amputation, as demonstrated in the published studies [5,16].

Evaluation of added S53P4 BG compared to traditional surgical treatment in septic osteoarthritis of the first MTP joint showed itself to be effective. During the one-year follow-up, patients with S53P4 BG needed no additional antibiotic therapy or surgical intervention. Bioactive Glass S53P4, when applied to the diabetic foot, showed itself to be a safe bone substitute biomaterial. Despite the small groups in our comparison, it seems that the addition of Bioglass enables greater stability and has fewer late complications, such as great toe deformities (e.g., hallux valgus).

A larger, prospective randomized study with a longer follow-up period is necessary to confirm the favourable role of S53P4 BG compared to segmental resection without bone substitutes.

## Figures and Tables

**Table 1:** Characteristics of study participants.

Characteristics	Group A	Group B	*p*
	S53P4 BG (*N* = 10)	(*N* = 12)	
Gender (male/female)	8/2	9/3	0.781
Age (mean ± SD)	62.1 ± 13.88	57.0 ± 14.69	0.416
Comorbidities			
Diabetic neuropathy	10	12	1
Arterial hypertension	7	5	0.369
Hyperlipidemia	4	3	0.652
Coronary artery disease	1	2	1
Chronic renal failure	0	1	1
Stroke	1	0	0.454
Rheumatoid arthritis	1	2	1
PAD * (La Fontaine)			
I	4 (40%)	6 (50%)	0.689
IIa	5 (50%)	5 (41.7%)	
IIb	1 (10%)	1 (8.3%)	

* PAD—Peripheral artery disease.

**Table 2:** Microbiological findings from bone sample cultures.

Isolated Pathogen, *N* (%)	Group A	Group B	*p*
Staphylococcus aureus methicillin-sensitive	5 (29%)	8 (33%)	ns
Staphylococcus aureus methicillin-resistant	1 (6%)	1 (4%)	ns
Beta hemolytic streptococcus	2 (12%)	1 (4%)	ns
Pseudomonas aeruginosa	0	1 (4%)	ns
Proteus spp	1 (6%)	0	ns
Coagulase negative staphylococci	1 (6%)	3 (13%)	ns
Other anaerobic bacteria	6 (35%)	10 (42%)	ns
Other bacteria	1 (6%)	0	ns
Total *N*	17	24	

## Data Availability

Not applicable.
